# Biosynthetic Modularity Rules in the Bisintercalator Family of Antitumor Compounds

**DOI:** 10.3390/md12052668

**Published:** 2014-05-09

**Authors:** Javier Fernández, Laura Marín, Raquel Álvarez-Alonso, Saúl Redondo, Juan Carvajal, Germán Villamizar, Claudio J. Villar, Felipe Lombó

**Affiliations:** Research Group BITTEN, Instituto Universitario de Oncología del Principado de Asturias (IUOPA), Universidad de Oviedo, C/Julián Clavería 7, Facultad de Medicina, Oviedo 33006, Spain; E-Mails: uo186966@uniovi.es (J.F.); marinla01@gmail.com (L.M.); rachelmartes13@gmail.com (R.A.-A.); redondo.saul@gmail.com (S.R.); UO223177@uniovi.es (J.C.); gvillami2002@yahoo.es (G.V.); cjvg@uniovi.es (C.J.V.)

**Keywords:** bisintercalator, antitumor, antibiotic, antiviral, non-ribosomal peptide, 3-hydroxy-quinaldic acid, quinoxaline-2-carboxilic acid, actinomycete, depsipeptide, thiodepsipeptide

## Abstract

Diverse actinomycetes produce a family of structurally and biosynthetically related non-ribosomal peptide compounds which belong to the chromodepsipeptide family. These compounds act as bisintercalators into the DNA helix. They give rise to antitumor, antiparasitic, antibacterial and antiviral bioactivities. These compounds show a high degree of conserved modularity (chromophores, number and type of amino acids). This modularity and their high sequence similarities at the genetic level imply a common biosynthetic origin for these pathways. Here, we describe insights about rules governing this modular biosynthesis, taking advantage of the fact that nowadays five of these gene clusters have been made public (thiocoraline, triostin, SW-163 and echinomycin/quinomycin). This modularity has potential application for designing and producing novel genetic engineered derivatives, as well as for developing new chemical synthesis strategies. These would facilitate their clinical development.

## 1. Antitumor Compounds from the Bisintercalators Family: The Origins

A handful of actinomycetal bioactive compounds from marine and terrestrial origins possess a very distinct chemical feature, allowing them to react in a very special way with DNA helix. All these compounds contain a cyclic structure formed by various amino acids. It is actually the result of a head to tail dimerization from two identical amino acid core chains generated by a non-ribosomal peptide synthetase (NRPS). Two identical planar bicyclic heteroaromatic moieties are also bound to this cyclic structure, which protrude at both distant positions from this symmetric peptide scaffold. These chromophore units are the starting moieties used during the assembly line of these NRPSs, and this is the reason to call these compounds bisintercalators [1,2]. The general structure is therefore a complex peptide scaffold plus two protruding bicyclic heteroaromatic chromophores. This is what makes it possible to firmly maintain both chromophores facing out from the peptide scaffold, deeply interacting with the DNA minor groove [3]. During this interaction, both chromophores are inserted in between the minor groove base pairs, unwinding and extending the DNA helix. This causes alteration of diverse cellular processes associated with replication and transcription that eventually will kill the affected cell [4].

**Scheme 1:**
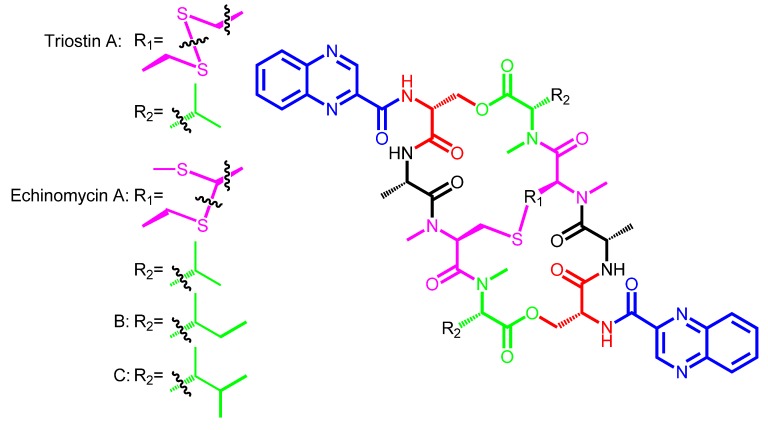
Chemical structures of triostin A and echinomycins/quinomycins A, B and C, showing the two different possibilities for intramolecular disulfide/thioacetal linkage.

Since the discovery of echinomycin in Germany in 1957 (produced by *Streptomyces echinatus*) (Scheme 1) and the same compound in Japan in 1961 (named quinomycin, produced by *Streptomyces* sp. 732), this family of bisintercalator natural products has been including new members at a very slow discovery rate. Nowadays, five producers of echinomycin are known (Table 1), which gives just an idea of how these biosynthetic pathways must be widely spread in nature [5,6,7,8,9]. 

**Table 1:** List of bisintercalator compounds, their producer actinomycetes and their known derivatives.

Compound	Natural Derivatives	Producer	Origin of Strain	Synthetic Derivaties
**SW-163C**	SW-163D	*Streptomyces* sp. SNA15896	Japan	−
	SW-163E			
	SW-163F			
	SW-163G			
**Triostin A**	−	*Streptomyces triostinicus*	Japan	TANDEM
		*Streptomyces lasaliensis*		SNAC-derivatives
		*Streptomyces aureus* s-2-210-L		
**Echinomycin A** (Quinomycin A)	Quinomycin B	*Streptomyces echinatus*	Angola, Japan	Ecolymicin C
	Quinomycin C	*Streptomyces lasaliensis*		1QN
	Quinomycin D	*Streptomyces* sp. KN-0647		2QN
	Quinomycin E	*Streptomyces* sp. 732		
		*Streptomyces griseovariabilis* subsp. *bandungensis*		
**Thiocoraline**	−	*Micromonospora marina* ML1	Mozambique Strait	Marburg-derivatives
		*Micromonospora marina* ACM2-092		Azathiocoraline
				NMe-Azathiocoraline
				Oxathiocoraline
**BE-22179**	−	*Streptomyces* sp. A22179	Japan	−
**Sandramycin**	−	*Nocardioides* sp. ATCC 39419	Mexico	−
**Quinaldopeptin**	−	*Streptoverticillium album* Q132-6	India	−
**Luzopeptin C**	Luzopeptin A	*Actinomadura luzonensis*	Philippines	−
	Luzopeptin B			
**Quinoxapeptin C**	Quinoxapeptin A Quinoxapeptin B	Unclassified nocardioform actinomycete	Alaska	−

In this respect, it is interesting to mention that spreading of this type of biosynthetic gene clusters between different microorganisms could be partly favored by the fact that some gene clusters coding for bisintercalator compounds are chromosomally encoded, but others are present in giant plasmids: the 520 kb pKSL plasmid from *S. lasaliensis* contains the echinomycin gene cluster [10,11]. 

However now, 57 years after the first discovery of a natural bisintercalator compound, only 21 different natural individual compounds are still known. Their structures have been extensively revised [12,13]. Most of these 21 bisintercalators are different from other ones in just small enzymatic modifications. This implies that some of them are natural intermediates found in other producers, or natural combinatorial biosynthesis examples produced by evolutionary shuffling of building blocks used by the enzymatic machinery of these complex biosynthetic systems (Table 2) [14,15]. 

**Table 2:** List of bisintercalators with their chemical architecture.

Compound	Chromophore	Amino Acid A	Amino Acid B	Amino Acid C	Amino Acid D	Amino Acid E
**(unknown ancestor X)**	3HQA	d-Ser	−	l-Ala	*N*-methyl-l-Cys	*N*-methyl-l-Val
					(disulfide bridge)	
**SW-163C**	3HQA	d-Ser	−	l-Ala	*N*-methyl-l-Cys	*N*-methyl-Norcoronamic acid
					(disulfide bridge)	
**SW-163D,E,F,G**	3HQA	d-Ser	−	l-Ala	*N*-methyl-l-Cys	*N*-methyl-Norcoronamic acid
					(thioacetal bridge)	
**Triostin A**	QXCA	d-Ser	−	l-Ala	*N*-methyl-l-Cys	*N*-methyl-l-Val
					(disulfide bridge)	
**Echinomycin A**	QXCA	d-Ser	−	l-Ala	*N*-methyl-l-Cys	*N*-methyl-l-Val
					(thioacetal bridge)	
**Thiocoraline**	3HQA	d-Cys	−	Gly	*N*-methyl-l-Cys	*N,S*-dimethyl-l-Cys
					(disulfide bridge)	
**BE-22179**	3HQA	d-Cys	−	Gly	*N*-methyl-l-Cys	*N*-methyl-dehydro-Ala
					(disulfide bridge)	
**Sandramycin**	3HQA	d-Ser	l-Pipecolic acid	Gly	Sarcosine	*N*-methyl-l-Val
**Quinaldopeptin**	3HQA	d-DABA	l-Pipecolic acid	Gly	Sarcosine	l-Pipecolic acid
**Luzopeptin C**	6-methoxy-3HQA	d-Ser	4-OH-Δ-piperazic acid	Gly	Sarcosine	β-OH-*N*-methyl-l-Val
**Quinoxapeptin C**	6-methoxy-QXCA	d-Ser	4-OH-Δ-piperazic acid	Gly	Sarcosine	β-OH-N-methyl-l-Val

This means that, on the one hand, our knowledge of the natural diversity of this family of compounds is restricted by the fact that only these 21 variants have been identified so far. Nowadays, under industrial-based screening processes devoted for isolation of new drugs (especially antibiotics, antitumor agents, antiparasitics and antivirals), extracts from new isolated microbe species are routinely scanned for already known compounds families. This routine attempts to assure that any interesting bioactivity in a giving extract will not render again scaffolds similar to known compounds. The reason is that known compounds (or similar ones) would be less interesting for patent/commercialization purposes [16]. Given the fact that this screening philosophy for new bioactive compounds will probably continue for many years, there is a real risk that new bisintercalators different to the current 21 known ones will never emerge in the literature as discarded “known-ones” during discovery processes. The main disadvantage from this is that our understanding of the evolutionary aspects and biosynthetic rules of this family of compounds will be more difficult to complete, or may be an impossible task.

On the other hand, these 21 known bisintercalators are produced from species belonging to distant taxonomical branches of the actinomycetes tree (Table 1). Five different genera (*Actinomadura*, *Micromonospora*, *Nocardioides*, *Streptomyces*, *Streptoverticillium* and another unclassified actinomycete isolated from the bark of a birch *Betula* species) have been described as producers, which belong to four distant families (*Thermomonosporaceae*, *Micromonosporaceae*, *Nocardioidaceae* and *Streptomycetaceae*) [17,18,19,20,21,22]. This taxonomical diversity and the fact that these producer microorganisms were obtained from seas and lands of Africa, Asia and America continents, as well as that four different compounds were isolated just in strains collected in a single country (Japan) (Table 1) implies that probably new producers exist in different ecosystems all over the planet, just waiting for someone to discover them. 

## 2. Structural Diversity and Modularity in the Bisintercalators Family: Suggestions of a Common Evolutionary Origin?

There is a nice structural variability found in these 21 known bisintercalators. It is composed by simple rules which dictate for example that all known natural bisintercalators are the result of the evolutionary use of just two chromophore subunits, the 3-hydroxy-quinaldic acid (3HQA) and the quinoxaline-2-carboxylic acid (QXCA) [15]. 

**Scheme 2:**
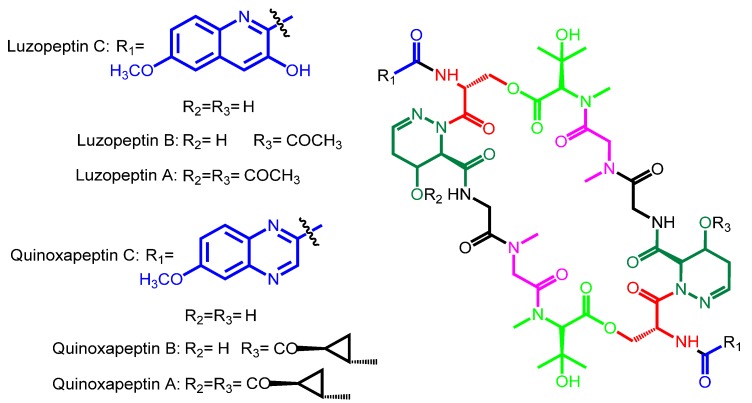
Chemical structures of luzopeptins A, B, C and quinoxapeptins A, B, C; where chromophores have been tailored to 6-methoxy derivatives.

Also, as another example of natural combinatorial biosynthesis in this family, these two types of chromophores are also decorated in a couple of cases by the action of a 6-hydroxylase and a methyltransferase acting on the resulting hydroxyl moiety. These two tailoring enzymes generate the corresponding 6-methoxy-3HQA and 6-methoxy-QXCA derivatives, found in luzopeptin and quinoxapeptin, respectively (Table 2, Scheme 2) [22,23]. With respect to amino acids core composition, all bisintercalators, as it has been described above, are composed of a short linear non-ribosomal peptide bound to the chromophore. This basic half subunit of the molecule is connected in a head-to-tail way to the same structure, creating a symmetric cyclic peptide scaffold [1,15].

**Scheme 3:**
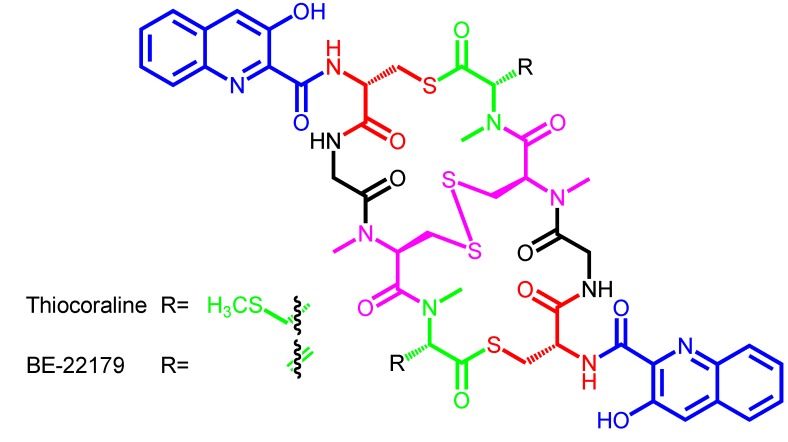
Chemical structures of thiocoraline and BE-22179, the only known two members containing a thiodepsipeptide structure.

**Scheme 4:**
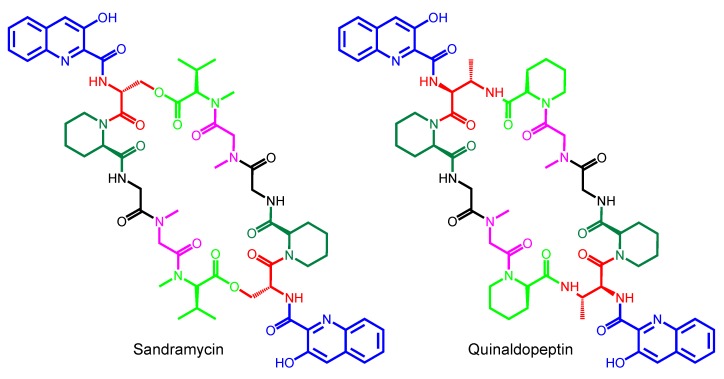
Chemical structures of sandramycin and quinaldopeptin, which belong to Major scaffold type (five amino acids in each half of the molecule).

Depending on the specific amino acid composition at both ends of this amino acid sequence (at both symmetric halves), the macrocycle structure is going to be connected by ester, thioester or amide linkage. This depends just on the presence of d-Ser, l-Cys or d-DABA (d-diaminobutyric acid) in the first amino acid position, respectively [21,24,25]. This will generate a cyclic depsipeptide (SW-163, triostin, echinomycin, sandramycin, luzopeptin, quinoxapeptin), thiodepsipeptide (thiocoraline, BE-22179, Scheme 3) or peptide (quinaldopeptin, Scheme 4) scaffold, accordingly [12,13].

**Figure 1:**
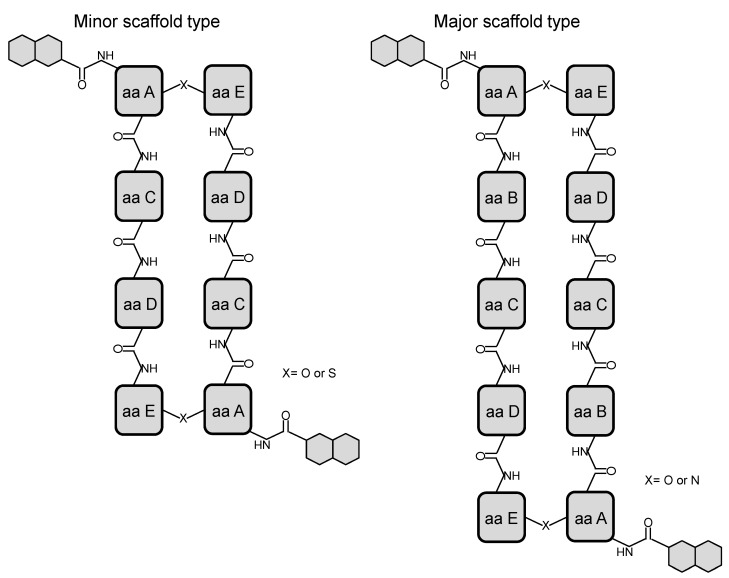
Minor and Major scaffold types in bisintercalator compounds.

**Scheme 5:**
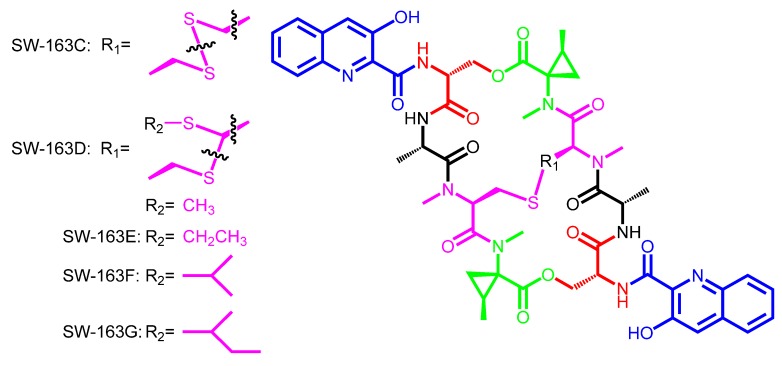
Chemical structures of SW-163C, D, E, F and G, showing the two possibilities for disulfide bridge or thioacetal bond.

**Figure 2:**
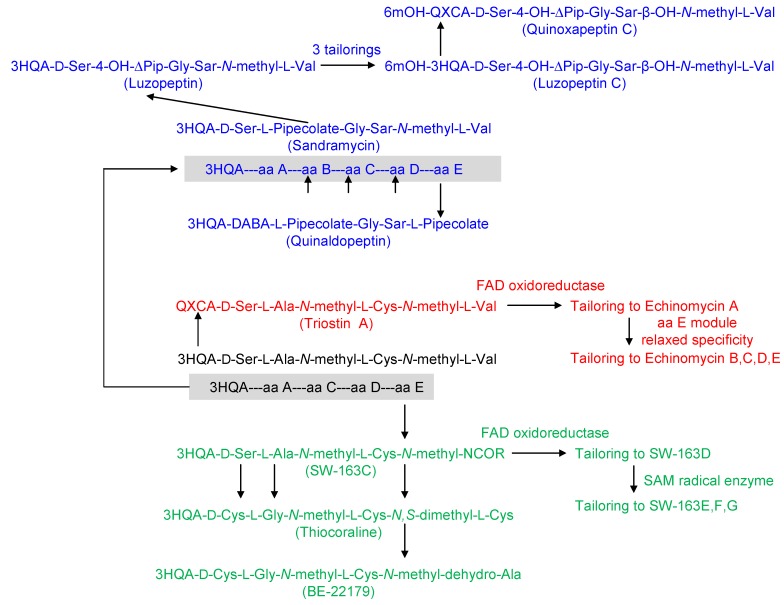
A hypothetical evolutionary tree on diversification of bisintercalators biosynthetic systems based on current known compounds.

Also, all bisintercalators belong to just two main biosynthetic scaffolds (Figure 1). Minor scaffold type contains four amino acids in each one of the molecule symmetric peptide half (Table 2). This type includes the SW-163 subgroup (Scheme 5), the triostin-echinomycin subgroup (Scheme 1), and the thiocoraline-BE-22179 subgroup (Scheme 3). The other type, Major scaffold, contains five amino acids in each one of the molecule symmetric peptide half. This other type includes the sandramycin-quinaldopeptin subgroup (Scheme 4) and the luzopeptin-quinoxapeptin subgroup (Scheme 2). Between each one of these five structural subgroups, the differences arise mainly due to the branched evolutionary acquisition of a different building block between its members (a different chromophore or amino acid) (Figure 2). It is noteworthy to mention that most of the described amino acids in this family of non-ribosomal peptide compounds are actually non-proteinogenic amino acids. These result from the epimerization, methylation, metabolism or cyclization of common amino acids. We found therefore examples as d-Ser or d-Cys, *N*-methyl-l-Cys, sarcosine or the rare cyclic building blocks *N*-methyl-norcoronamic acid, l-pipecolic acid or 4-OH-Δ-piperazic acid (Table 2).

Interestingly, the identity of the amino acid placed at position D (Table 2) in any giving bisintercalator will give rise to another structural property in this family of compounds, which is just derived from the identity of these two D moieties in the symmetrical head-to-tail peptide scaffold. These faced amino acids can be two l-Cys derivatives (as in all the bisintercalators from the Minor scaffold type) (Scheme 1, Scheme 3 and Scheme 5), and in this case, the final molecule will contain a disulfide bridge connecting both halves of the symmetrical compound. Due to the existence in some of these gene clusters of genes coding for some special tailoring enzymes, in some cases, this disulfide bridge (as in triostin, Scheme 1) will be converted into a thioacetal bond (as in echinomycin) by the action of a SAM methyltransferase. These modifications increase the structural diversity and modularity of this family, as formation of the interconnecting bridge/bond generates the existence of two sub-rings in the general scaffold [26]. Also, in the case of SW-163 subgroup, the tailoring goes further and the thioacetal is further modified by diverse alkylations, ranging from methyl to sec-butyl moieties (Scheme 5) [27,28]. 

However, in all bisintercalators from the Major scaffold type, the amino acid placed at D position is sarcosine, and as this moiety does not contain sulfhydryl groups, it is not possible to establish a disulfide bridge once the biosynthesis has been completed. All these four compounds, therefore, maintain just a macrocycle in the general scaffold, as in the sandramycin-quinaldopeptin subgroup (Scheme 4) and in the luzopeptin-quinoxapeptin subgroup (Scheme 2).

In Table 2, we have included the hypothetical chemical architecture for the biosynthetic building blocks of an unknown bisintercalator X (first row). This is a way to hypothesize how the different sequential evolutionary changes could have been taking place in order to explain the current (narrow) structural spectrum of known bisintercalators. This unknown putative ancestor contains 3HQA as chromophore, as this type of compounds show higher diversity with respect to amino acids composition (and therefore we think this would indicate extra evolutionary time). Also, enzymatic transformations rendering 3HQA are simpler than in the QXCA case. There are high identities and similarities between common 3HQA and QXCA biosynthetic steps, ranging from 61%/74% (as for TioK/Qui18) to 31%/46% (as for TioF/TrsC). This could support a common ancestor for both pathways. Also, at NRPS level, sequence identities/similarities are high between triostin and echinomycin (74%/81% for TrsJ/Ecm6, 79%/86% for TrsI/Ecm7), or triostin/echinomycin and SW-163C (75%/81% for TrsJ/Swb16, 70%/77% for Ecm6/Swb16, 73%/82% for TrsI/Swb17). However, these identities/similarities are lower when comparing triostin/echinomycin and thiocoraline (62%/72% for TrsJ/TioR, 60%/70% for Ecm6/TioR, 65%/77% or TrsI/TioS, 63%/75% for Ecm7/TioS). This is in accordance with our current hypothesis, where triostin/echinomycin are closer relatives of SW-163C, yet thiocoraline would be the result of a later evolution from SW-163C and therefore it is more distant than triostin/echinomycin. 

We can start with this unknown compound X containing the basic sequence 3HQA-d-Ser-l-Ala-*N*-methyl-l-Cys-*N*-methyl-l-Val (chromophore plus four amino acids: A-C-D-E). This could be dimerized and cyclized maintaining a disulfide bridge, if a corresponding thioesterase domain would have been recruited for the dimerization. Changing amino acid E specificity towards norcoronamic acid (which has been actually described as deriving from l-Val), would have rendered SW-163C, where this uncommon amino acid is *N*-methylated by the NRPS [27]. Acquiring the methyltransferase activity required for transformation of a disulfide bridge into a thioacetal bond would then allowed the generation of SW-163D, E, F and G scaffold. These derivatives vary also in the presence of different moieties (methyl, ethyl, propyl and sec-butyl) attached to the thioacetal bond (Figure 2). 

From a pathway devoted for SW-163C biosynthesis, alterations in amino acids A and C specificity towards d-Cys and Gly respectively, and then in amino acid E specificity towards *N*,*S*-dimethyl-l-Cys would have rendered a system able to produce thiocoraline. This pathway comes from marine *Micromonospora* species (Mozambique Strait), whereas SW-163C comes from terrestrial *Streptomyces* species (Japan) [18,29]. If amino acid E NRPS adenylation domain, however, would have changed its specificity towards *N*-methyl-dehydro-Ala, the formed compound would have been BE-22179. 

However, from the unknown bisintercalator X, a biosynthetic change consisting in changing the enzymatic machinery for 3HQA pathway transformation towards QXCA would have generated triostin A. Its further conversion towards echinomycin A needs the assistance of a tailoring methyltransferase in charge of thioacetal bond generation (from the disulfide bridge present in triostin A). Echinomycins/quinomycins B, C, D and E generation has been already explained as a consequence of the presence in the culture medium of diverse branched amino acids as isoleucine, which indicates a relaxed specificity for the amino acid placed at position E in this enzymatic assembly line [30,31]. 

Finally, starting also from the unknown bisintercalator X, changing the specificity in this NRPS system for amino acids C and D towards Gly and sarcosine, respectively, and acquisition in the new NRPS system of an extra biosynthetic module for amino acid placed at position B (l-pipecolic acid), would have rendered a system for generation of sandramycin. From this stage, two changes in amino acids A and E specificities towards D-DABA and l-pipecolic acid, respectively, would have generated quinaldopeptin. From the sandramycin system, changing the amino acid B specificity towards 4-OH-Δ-piperazic acid (instead of l-pipecolic acid) and gain of two enzymes for tailoring chromophore 3HQA towards 6-methoxy-3HQA, as well as tailoring the amino acid E (*N*-methyl-l-Val) towards β-OH-*N*-methyl-l-Val, would have rendered luzopeptin C. In a similar way, acquisition in the luzopeptin system of the machinery for QXCA generation (instead of 3HQA), would have rendered 6-methoxy-QXCA as chromophore. This means that quinoxapeptin C has been generated probably as evolution from luzopeptin. 

Both, luzopeptin and quinoxapeptin systems, possess extra degrees of tailoring structural diversification. In luzopeptin B, the 4-hydroxy group at one 4-OH-Δ-piperazic acid residue is acetylated, whereas in luzopeptin A both 4-hydroxyl groups are acetylated [17,32]. The equivalent 4-OH-hydroxyl groups of quinoxapeptin A are also tailored with 2-methyl-cyclopropane carboxylic acids, whereas in quinoxapeptin B one of them contains acetate instead [22,33]. 

Isolation of new bisintercalator compounds from new natural actinomycetes samples would shed light on the evolution of these complex NRPS systems. 

## 3. Modular Biosynthesis in the Bisintercalators Family

All bisintercalator compounds follow the same modular biosynthetic scheme. Under this modular assembly line, a chromophore starter moiety (3HQA or QXCA), is activated by a ligase to the corresponding AMP-derivative, and it is bound to a peptidyl-carrier-protein (PCP) on a NRPS system. 

Then, four different NRPS modules (for the Minor scaffold type compounds) or five different ones (for the Major scaffold type molecules) carry out a step by step incorporation of the corresponding activated amino acids (four or five, respectively). Each NRPS module carries out an elongation cycle during this assembly line, incorporating a specific amino acid into the final compound structure. The basic module architecture comprises three domains [33]: an adenylation domain (A, about 550 amino acids long), which selects and activates an specific amino acid into its AMP-derivative, a peptidyl-carrier protein domain (P, about 80 amino acids), which binds the activated amino acid to its 4′-phosphopantetheine prosthetic group via a thioester bond, and a condensation domain (C, about 450 amino acids) which generates a new peptide bond between two aminoacyl adenylated residues located on consecutive P domains [34,35,36,37,38]. Also, there is a colinearity rule between the NRPS modules organization and the structure of the final compound [39,40].

In most bisintercalators, these amino acids are non-proteinogenic. Therefore, in the corresponding activation and transfer NRPS module, distinctive domains are necessary (some of these as free proteins), showing epimerase, hydroxylase, *N*- or *S*-methyltransferase activities. Some other amino acids are simple (d-DABA) or complex (*N*-methyl-norcoronamic acid, l-Pipecolic acid, 4-OH-Δ-piperazic acid) derivatives from diverse common amino acids (l-Thr, l-Val, l-Lys, l-Glu). This implies specific metabolic pathways in order to create these elongation units that will be further incorporated by the corresponding NRPS module. Once the corresponding linear non-ribosomal peptide has been completely formed, it is transferred to the last NRPS-domain, a thioesterase domain (TE), which then carries out the head-to-tail dimerization to an identical linear peptide, as well as the cyclization of the final molecule. The presence of such uncommon amino acids provides extra protection to the final bisintercalator against proteolytic cleavage (proteases).

In some cases, the position E in the linear peptide core is harboring *N*-methyl-l-Cys. Then, a spontaneous reaction or specific tailoring enzymes are in charge of generation of a disulfide bridge (Scheme 1, Scheme 3 and Scheme 5) once the free cyclic non-ribosomal peptide has been dimerized and released from the NRPS assembly line. This bridge can be also further tailored to a thioacetal bond, which is also prone to be acylated in some cases (Scheme 1 and Scheme 5). 

Also, in some Major scaffold type bisintercalators, a 6-methoxy tailoring at chromophores exists (luzopeptins and quinoxapeptins) (Scheme 2). 

### 3.1. Biosynthesis of the Chromophore Moieties

The two chromophore types present in known bisintercalators (3HQA and QXCA) derive from l-Trp. This has been shown with feeding experiments and also with fluoro-l-Trp labeling in new derivatives [41,42]. Based on current experimental evidence; l-Trp is first bound to a small monomodular NRPS protein formed by an A-T module, as in TioK (from the thiocoraline gene cluster), Swb11 (SW-163 gene cluster), TrsR (triostin) and Ecm13/Qui18 (echinomycin). The function of these adenylating enzymes needs the structural assistance of small MbtH-like proteins encoded in the corresponding gene clusters (TioT, Swb18, TrsH, Ecm8, Qui5). Then, a hetero-tetramer is formed with two subunits of each enzyme type. Absence of MtbH-like counterpart inhibits this first l-Trp reaction [9,43,44]. 

**Scheme 6:**
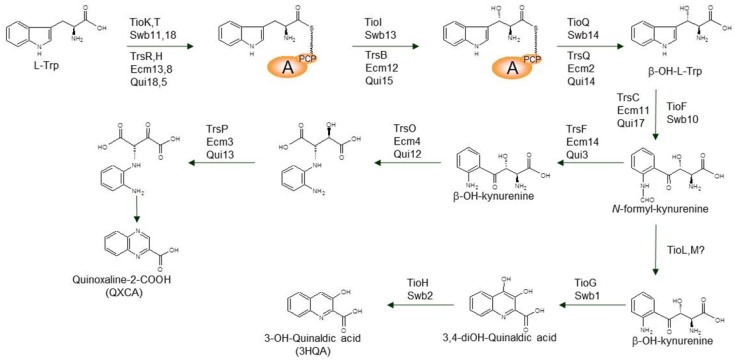
Biosynthetic steps towards 3HQA and QXCA.

This attached l-Trp (Scheme 6) is then β-hydroxylated by the action of a cytochrome P_450_ (TioI, Swb13, TrsB, Ecm12, Qui15). The resulting β-hydroxy-l-Trp is then released by the action of a free type II thioesterase (TioQ, Swb14, TrsQ, Ecm2, Qui14) [43,44,45]. Indole ring opening at the β-hydroxy-l-Trp is achieved by the action of a Trp-2,3-dioxygenase (TioF, Swb10, TrsC, Ecm11, Qui17), generating *N*-formyl-β-hydroxykynurenine [46]. Loss of this formyl moiety can be spontaneous, or other enzymes could be involved in this (TioL, TioM, TrsF, Ecm14, Qui3) [15,43]. The resulting molecule, β-hydroxykynurenine, has been used in successful feeding experiments, demonstrating its existence in this biosynthetic pathways [47]. Up to this point, these l-Trp modifications are somehow similar to those produced during formation of kynurenine in liver and other vertebrate organs, where it is a key intermediate of NADH [15,48].

β-hydroxykynurenine is a common intermediate in these two biosynthetic pathways [47]. This compound is the substrate of an aminotransferase (TioG, Swb1), which generates the bicyclic heteroaromatic intermediate 3,4-dihydroxy-quinaldic acid (Scheme 6), which is further transformed by the action of an oxidoreductase (TioH, Swb2) into 3HQA [15].

For the QXCA chromophore type, β-hydroxykynurenine needs a further oxidative rearrangement catalyzed by TrsO/Ecm4/Qui12 (Scheme 6). This is followed by the oxidation of the generated secondary alcohol, which is carried out by TrsP/Ecm3/Qui13. A final cyclization and spontaneous aromatization render QXCA [43,45]. 

These two chromophores are later incorporated (as the starting units) to the corresponding biosynthetic linear assembly line. This incorporation takes place upon their activation by adenylation enzymes. This has been demonstrated by feeding and *in vitro* enzymatic experiments [49,50]. 

### 3.2. Biosynthesis of Distinctive Amino Acids

Some of the non-proteinogenic amino acids found in bisintercalator compounds are just the result of the existence of *N*-methyltransferase or *S*-methyltransferase domains in specific D and E NRPS modules (Table 2), where they carry out the generation of amino acids as *N*-methyl-l-Cys, *N*-methyl-l-Val, *N*,*S*-methyl-l-Cys, sarcosine (*N*-metyl-Gly) and *N*-methyl-dehydro-Ala. Also, the existence of d-Cys or d-Ser at the A position of these compounds (Table 2) is just the result of the presence of an epimerase domain at the corresponding NRPS module. 

**Scheme 7:**
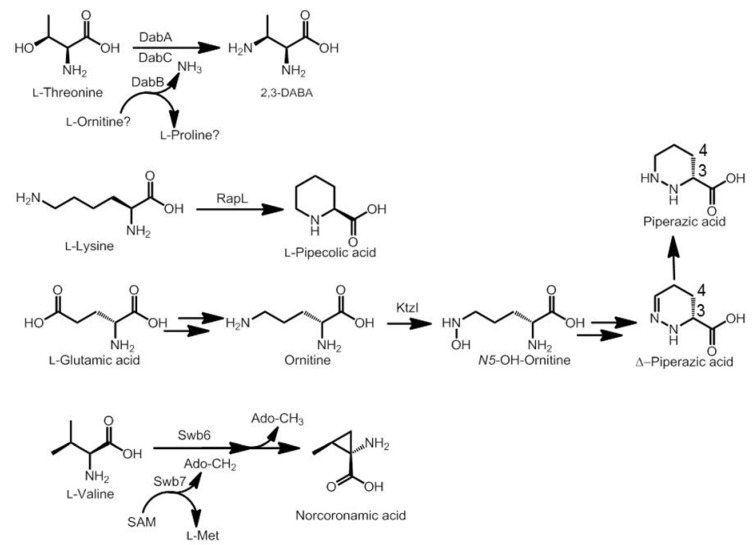
Biosynthesis of non-proteinogenic amino acids present in bisintercalator compounds.

However, other amino acids present in this family of compounds require complex biosynthetic pathways involving diverse enzymes acquired by the corresponding producing microorganism. This is the case of d-DABA (position A), 4-OH-Δ-piperazic acid (position B), l-pipecolic acid (positions B or E) and *N*-methyl-norcoronamic acid (position E). These uncommon amino acids require also the acquisition (by the counterpart NRPS module) of an adenylation domain specific for these rare moieties.

d-DABA is an epimerization of l-DABA, resulting from the existence of the corresponding epimerization domain in the NRPS assembly line. l-DABA biosynthesis (Scheme 7) is not yet fully understood, but it seems that it is derived from l-Thr by the action of three sequential enzymes (DabABC, DABA synthase), whose coding genes have been identified in gene clusters not related to bisintercalators (friulimycin from *Actinoplanes friuliensis*, laspartomycins from *Streptomyces viridochromogenes* ATCC 29814) [51,52]. DabA has similarity to pyridoxal phosphate (PLP)-dependent cysteine synthases, as the aminotransferase VioB from the viomycin pathway (where diaminopropionic acid is formed). DabB is similar to the deaminase VioK. Similar enzymes to DabA and DabB have been described in the siderophore staphyloferrin B biosynthetic gene cluster from *Staphylococcus aureus* (SbnAB) [53]. Experimental evidence from the mureidomycin biosynthetic pathway in *Streptomyces flavidoviridens* SANK 60486 clarifies that transformation of l-Thr into l-DABA involves a β-replacement reaction (OH in l-Thr towards NH_2_ in l-DABA), involving a PLP-dependent enzyme (DabA homologue) which transfers to l-Thr an amino group generated by a deaminase (DabB homologue) [54]. Further structural studies in DABA synthase are expected. 

l-pipecolic acid biosynthesis has been described in the rapamycin biosynthetic pathway (*Streptomyces hygroscopicus*), where it is carried out by RapL (l-Lys cyclodeaminase) in just one enzymatic step (Scheme 7) [55]. Homologues to this enzyme have been also described in FK-506 (FkbL, *Streptomyces* sp. *kMA6548*), virginiamycin S (VisC, *Streptomyces virginiae*) and tubulysin (TubZ, *Angiococcus disciformis*) pathways [56,57,58]. Sandramycin and quinaldopeptin gene clusters, must therefore contain *rapL* homologues, as these bisintercalators contain l-pipecolic acid. 

4-OH-Δ-piperazic acid is found in luzopeptins and quinoxapeptins (Table 2). The basic structure of this moiety is Δ-piperazic acid. This 4-hydroxy compound is incorporated *in vitro* by the NRPS assembly line of these antitumor agents*.* This demonstrates that the 4-hydroxy group is not a final tailoring modification of the finished bisintercalator [33]. Anyway, Δ-piperazic acid is a very uncommon non-proteinogenic amino acid, whose presence in natural products has been recently nicely revised [59]. Its biosynthesis has been described as an intermediate of piperazic acid in the sanglifehrin biosynthetic pathway, deriving from l-Glu. This biosynthetic origin has been unambiguously demonstrated by *in vitro* experiments using enzymes from the kutzneride biosynthetic pathway (from *Kutzneria* sp. 744) [60,61]. In these experiments, the l-Glu could be converted to ornithine as part of the primary bacterial metabolism, which is used as substrate by the hydroxylase KtzI, generating *N*5-hydroxy-ornitine (Scheme 7). This secondary metabolism intermediate is incorporated in feeding experiments into the final kutzneride compound. *N*5-hydroxy-ornithine must be converted into Δ-piperazic acid after oxidation to its nitroso derivative. Finally, its cyclization carried out by still unidentified enzymes [61].

Norcoronamic acid is present only in SW-163 compounds, at the position E of the linear peptide core. It appears there as *N*-methyl derivative, due to the presence in this NRPS module of a probable *N*-methyltransferase domain. Current hypothesis explains that its biosynthesis derives from l-Val by the action of a PLP-dependent aminotransferase (Swb6) and a radical SAM methyltransferase (Swb7), generating this cyclopropane derivative (Scheme 7) [27].

### 3.3. Use of the Chromophore Moiety as Starter in the NRPS Assembly Line

**Scheme 8:**
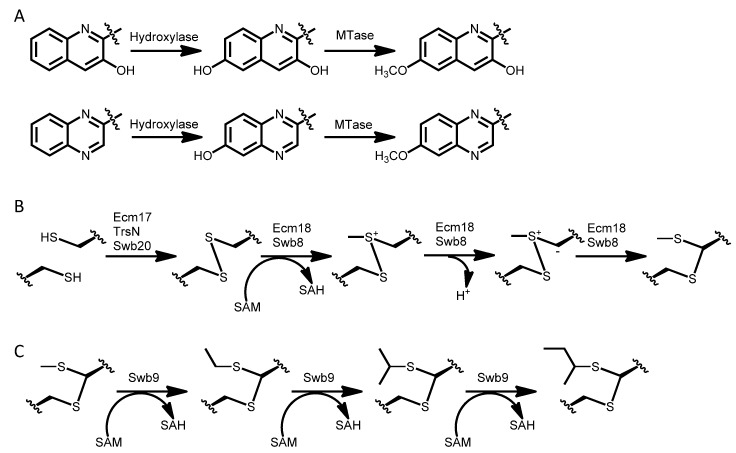
Tailoring modifications during the final steps of the biosynthesis of bisintercalators. (**A**) Methoxy group formation in chromophores; (**B**) Thioacetal bond formation; (**C**) Iterative alkylation in SW-163E, SW-163F and SW-163G.

Once the corresponding 3HQA or QXCA chromophore has been formed in the producing microorganism, an AMP-ligase (TioJ, Swb12, TrsA, Ecm1 and Qui16) activates this moiety (Scheme 8). The activated chromophore is then attached to a free peptidyl-carrier domain. In thiocoraline, this P domain is present as an independent enzyme coded in the cluster, TioO. However, in other sequenced clusters, there is not such an specific enzyme, and the fatty acid biosynthesis FabC protein (an acyl-carrier protein) has been involved in this linkage [15,62]. 

**Figure 3:**
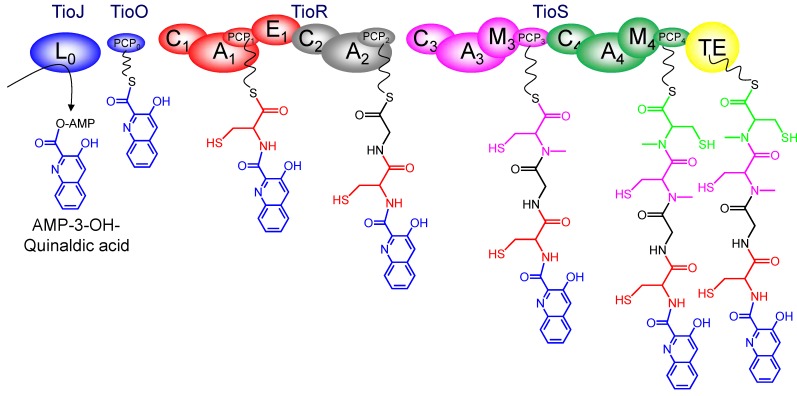
Non-ribosomal peptide synthetase (NRPS) assembly line in bisintercalator natural compounds (thiocoraline version). L: Ligase domain, PCP: Peptidyl carrier protein domain, C: Condensation domain, A: Adenylation domain, E: Epimerization domain, M: Methyltransferase domain, TE: Thioesterase domain.

The P-bound chromophore is then the starting point for the step by step incorporation of four (Minor scaffold type) or five (Major scaffold) specific amino acids, by means of the NRPS assembly line (Figure 3). 

Although the amino acid composition of the final molecule is different in the five different sequenced gene clusters (thiocoraline, triostin, echinomycin, SW-163, quinomycin), however, the modular structure of these NRPSs is exactly the same (Scheme 8) [9,15,26,27]. This NRPS includes two proteins, each one containing two modules. The first module possesses the C-A-P-E domains able to incorporate the amino acid at position A and to carry out its epimerization (to d-Cys or d-Ser). The second module possesses a C-A-P structure, and in these five pathways it will incorporate Gly or l-Ala accordingly. The third module contains C-A-M-P domains, and it is responsible for the incorporation of l-Cys and its conversion into *N*-methyl-l-Cys. The fourth module has a C-A-M-P-TE structure and it is responsible for the incorporation (including its *N*-methylation) of *N*-methyl-norcoronamic acid, *N*-methyl-l-Val or *N*-methyl-l-Cys (which will be later converted into *N*,*S*-methyl-l-Cys by the probable action of TioN).

It is expected that future sequencing of other Minor scaffold type bisintercalators gene clusters will show a similar NRPS structure. Sequencing of the Major scaffold type gene clusters will demonstrate the existence of an extra NRPS module placed at position B (right after the first described module), responsible for activation and incorporation of l-Pipecolic acid (sandramycin, quinaldopeptin) or 4-OH-Δ-piperazic acid (luzopeptin, quinoxapeptin).

### 3.4. Dimerization, Cyclization and Scission of the NRPS Scaffold

All sequenced bisintercalator gene clusters possess a terminal thioesterase (TE) domain placed at the C terminus of the last NRPS assembly line modules. This TE domain is responsible for the intramolecular head-to-tail cyclization of the corresponding macrocyclic peptide, releasing it from the NRPS enzyme. 

The thiocoraline TE domain has been independently expressed and used *in vitro* against an array of activated tetrapeptides by using SNAC-thiophenol activation (*N*-acetylcysteinamine). A plethora of diverse thiocoraline-like molecules have been generated in this way. This shows that the d-stereochemistry found at amino acid placed at position A in all bisintercalators is needed in order to accomplish dimerization and cyclization instead of hydrolysis [63]. In a similar way, methylated l-Cys at position E is also required in order to accomplish cyclization, since the presence of only l-Cys causes hydrolysis. This TE domain can also give macrolactonization instead of macrothiolactonization if the amino acid at position A is d-Ser instead of d-Cys. The echinomycin TE domain has been also tested against several SNAC-substrates and it has been demonstrated to have a broad substrate spectrum [64]. 

**Figure 4:**
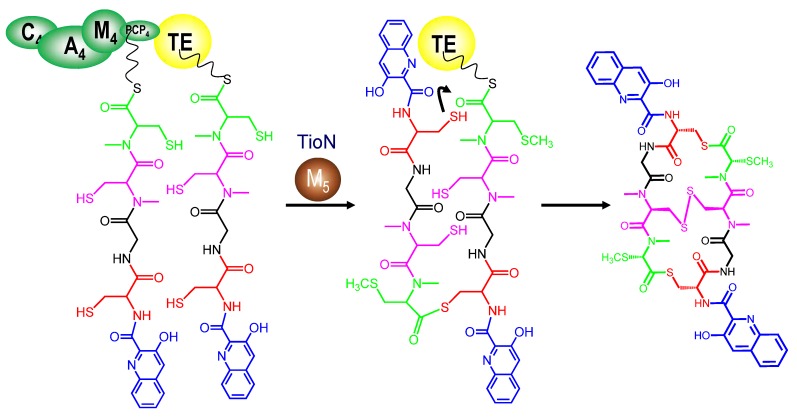
Cyclization carried out by thiocoraline thioesterase NRPS domain (TE) after the action of TioN *S*-methyltransferase (M_5_). C: Condensation domain, A: Adenylation domain, PCP: Peptidyl carrier protein domain.

The macrolactonization type depends on the amino acids present at positions A and E in the corresponding linear tetra- or penta-peptide core chain. Most bisintercalator compounds have ester linkages after cyclization, as they possess d-Ser at position A (Table 2). However, quinaldopeptin possesses d-DABA at this position, and it is the only bisintercalator with an amide ring closure. Finally, thiocoraline and BE-22179 contain d-Cys in this position, and these molecules are macrolactonized via a thioester bond (Figure 4). 

### 3.5. Tailoring Enzymes

Two Major scaffold type bisintercalators (luzopeptin and quinoxapeptin) possess tailored 6-methoxy-chromophores. Interestingly, the tailored chromophores in these two compounds belong to both types: 6-methoxy-3HQA in luzopeptins and 6-methoxy-QXCA in quinoxapeptins (Scheme 2). This implies that probably they are the result of the incorporation into these producer microorganisms of two enzymes: A 6-hydoxylase and a methyltransferase able to modify the 6 position of the corresponding chromophore. Therefore, this must be a late step (tailoring) in the biosynthesis of these two types of bisintercalators. 

Some bisintercalators harboring at position D a *N*-methyl-l-Cys moiety possess an oxidoreductase enzymatic activity able to generate between these two facing amino acids a disulfide bridge (triostin, echinomycin, SW-163). Ecm17/TrsN/Swb20 have been proposed as the corresponding FAD-dependent oxidoreductases for this reaction [65]. Surprisingly, some other disulfide bridge oxidoreductases, such as GliT (from a gliotoxin-producing fungus), are able to recognize and carry out this reaction on these bisintercalator intermediates [43,66,67]. No enzyme appears to carry out this reaction during thiocoraline biosynthesis. This opens the door to a spontaneous reaction in this case, or to the existence of an oxidoreductase outside the thiocoraline gene cluster in *Micromonospora* sp. ML1 able to carry out this disulfide bridge formation (Scheme 8) [15]. 

Disulfide bridges can also be converted into a thioacetal bond in some of the biosynthetic pathways (SW-163D-G, echinomycin). This has been described as the result of the enzymatic activity of the SAM methyltransferase Ecm18/Swb8, which carries out a methyl transfer, followed by deprotonation and rearrangement (Scheme 8) [27]. 

It is interesting the existence of diverse alkylations on the thioacetal bridge of SW-163D to G. The SAM radical enzyme Swb9 seems to carry out an iterative methylation, generating the methyl-, ethyl-, *i*-propyl- and *sec*-butyl-moieties that appear in these compounds, respectively (Scheme 8) [27].

### 3.6. Resistance and Secretion Enzymes

As bisintercalators are powerful DNA-binding molecules, it is expected that at least some of the resistance enzymes present in the producer microorganism may be counteracting this lethal activity. Several ABC-transporters (TioC-D, Swb4-5, TrsD-E, Qui1-2) are in charge of transmembrane exportation for these toxic molecules [9,15], therefore avoiding high concentrations of the bisintercalator in the producer actinomycete cytoplasm [26,27]. These canonical ATP-binding-cassettes transporters are formed by a transmembrane permease (which exports the compound to the outside of the cell) and an ATPase (which hydrolyzes ATP in order to get energy for the permease function), respectively. 

In the thiocoraline producer, the enzyme TioX is a tetrameric protein which captures and immobilizes thiocoraline in a similar way as the bleomycin resistance protein BlmA, therefore avoiding its bisintercalation activity on chromosomal DNA [68,69]. Also, in the sequenced gene clusters there are genes coding for UvrA-like proteins (TioU, Swb15, TrsM, Ecm16 and Qui10). These enzymes share similarity with MtrX from the mithramycin gene cluster and DrrC from the doxorubicin pathway. They probably repair DNA damages caused by bisintercalation in the producing bacteria chromosome [15].

## 4. Bioactivity of Bisintercalator Compounds

### 4.1. Binding to the DNA Minor Groove

In 1974, Waring and Wakelin, using viscometric techniques, demonstrated that echinomycin binds in a bisintercalative way to the DNA helix, inhibiting RNA polymerase-dependent transcription and other processes [70,71]. Since then, diverse techniques as DNase I footprinting, NMR, X-ray crystallography and electrospray ionization tandem MS have been used in order to establish the DNA sequence preferences of most of the known bisintercalators [72,73,74,75,76,77,78]. These experiments showed that this bisintercalation is carried out at the DNA minor groove by inserting the two planar heteroaromatic ring chromophores around two base pairs, like in a Mexican *taco-*like structure [79,80]. These experiments were also carried out using triostin, showing that the amino group orientation at the amino acid placed at position A, the one which binds to each chromophore moiety, protrudes these chromophores in a way that both heteroaromatic moieties are maintained parallel and oriented 90º with respect to the peptide scaffold, around the corresponding two base pairs [81,82]. Optical tweezers techniques have also shown that forces over 10 pN applied to the DNA-triostin complex cause dissociation of triostin and rebinding to the minor groove, but encompassing just one base pair [83]. 

The preference for 5′-CG base pairs or 5′-AT ones is not dictated by the type of chromophore moiety, but for a complex array of hydrogen bonds and other van der Waals forces established along the whole bisintercalator molecule (chromophore and peptide scaffold together) and the DNA minor groove bases [84,85]. This type of targeting towards DNA minor groove explains the antitumor and antimicrobial bioactivities found in all family members, as this bisintercalation blocks core cell processes as those dependent on RNA polymerase, therefore inhibiting transcription [86,87]. 

SW-163 compounds bind preferably to 5′-CG bases, as in the case of echinomycin and triostin, and binding strength to this 5′-CG pair is influenced by flanking sequences. For example, the preferred DNA region for echinomycin is 5′-ACGT [3,88]. Thiocoraline and BE-22179, which belong also to the Minor scaffold type as the previous compounds, containing different peptide scaffold, do not have a determined specificity for 5′-CG or 5′-AT sequences, although in thiocoraline a small preference for 5′-CG has been showed by using fluorescence melting, another proof of the importance of the peptide scaffold in this DNA minor groove interaction [75,89]. 

Sandramycin A binds preferably to 5′-AT sequences flanked by CG base pairs [90]. This DNA binding activity has been studied with various chromophore derivatives of these molecules, showing that an increase in the chromophore hydrophobicity (as 3HQA lacking the phenolic hydroxyl group) enhances the DNA binding capability [78,91]. DNA binding is 10 times more effective in sandramycin and in luzopeptin (both Major scaffold members) than in echinomycin. This means that probably, peptide scaffold enlargement enhances the DNA binding capability [92]. At this respect, it is noteworthy to mention that experiments using just the sandramycin peptide scaffold have demonstrated a high binding affinity to DNA minor groove [78]. Luzopeptin A and quinoxapeptin A (Major scaffold members), show also a preference for 5′-AT sequences [93,94,95]. Surprisingly, some Major scaffold members have been described also to carry out a bisintercalation between two different DNA helixes (each chromophore binding to a different DNA minor groove) as well as the canonical intramolecular DNA bisintercalation. This is favored when both chromophores are in *trans* configuration with respect to the peptide core [23,96]. 

### 4.2. Biological Activities

All bisintercalators show a general antitumor activity, which is greater in the Minor scaffold group [97,98]. Together with this biological effect, which is mainly derived from the DNA minor groove binding capabilities, all these compounds have shown strong antibacterial activities against Gram positive bacteria, also due to DNA binding activity. They are inactive against most Gram negative bacteria due to their huge size. This prevents these molecules from passing through their outer membrane [20]. For some members, inhibition of some cellular or viral specific enzymes has been also described [22].

SW-163 compounds are strong *in vitro* antitumor agents against a variety of cancer cell lines. The potency increases from the disulfide bridged SW-163C towards the other thioacetal and alkylated thioacetal members (SW-163D to G). Longer alkyl moieties cause a stronger antitumor effect. SW-163G shows also strong inhibition against the skin pathogens *Staphylococcus aureus* and *Erysipelothrix rhusiopathiae* [99]*.*

Echinomycins are potent antitumor agents. They are also active against many pathogenic Gram positive bacteria, as *S. aureus* (including methicillin resistant strains), *Streptococcus pneumoniae*, *Listeria monocytogenes* and *Enterococcus faecalis* (including vancomycin-resistant strains) [100,101,102,103]*.* As antiviral, it inhibits influenza virus multiplication and two enzymes from HIV-1 (Tat and reverse transcriptase) [89,104,105]. Echinomycins have demonstrated also a good activity against the protozoans *Plasmodium falciparum* and *Entamoeba histolytica* [100,106]. A second antitumor activity from these compounds lies in the selective inhibition of the Hypoxia Inducible Factor-1 (HIF-1), which usually binds to Hypoxia Response Elements (HRE) [107]. These HREs are placed in eukaryotic promoters which control genes involved in cellular survival, glycolysis, angiogenesis, migration and cellular invasion of cancer cells. HREs are very important targets for cancer therapy [108,109,110]. HRE are also important in non-tumor cell processes, as during heterotopic bone formation (after surgery or trauma), which depends also on hypoxia. Echinomycin has demonstrated a beneficial decrease of heterotopic bone formation in a murine Achilles tendon surgery model [111]. Also, HIF-1 activates VEGF (vascular endothelial growth factor) and therefore the multiplication of endothelial cells in blood vessels. In this respect, echinomycin has been tested in diminishing restenosis and thrombus formation after stent surgery [112]. 

Thiocoraline is active against human breast cancer, non-small-cell lung carcinoma, colon, renal and melanoma cancer cell lines *in vitro* and in *in vivo* xenografts [75,113]. Together with the strong DNA minor groove binding, this antitumor compound is also a strong inhibitor of DNA polymerase α subunit [75,114]. It inhibits a wide array of Gram positive pathogenic bacteria [18]. The other member of this subgroup, BE-22179, differs only in the amino acid placed at position E. BE-22179 shows also good antitumor activity against leukemia and gastric carcinoma, as well as against Gram positive pathogenic bacteria (*S. aureus*)*.* BE-22179 possesses a strong topoisomerase II inhibitory activity (over 200 and 300 times more potent than echinomycin and etoposide, respectively) [20,115]. 

Sandramycin is bactericidal against diverse Gram positive bacteria (*S. aureus*, *Bacillus subtilis*, *E. faecalis*), and also a good *in vitro* antitumor compound, with about 200 times stronger activity than echinomycin. Its *in vivo* antitumor capacities in a murine leukemia model are modest. This compound inhibits the HIV-1 reverse transcriptase [19,78]. Quinaldopeptin has about the same bioactivities as sandramycin, but it also shows good bioactivity against the fungus *Cryptococcus neoformans* [21]. 

Luzopeptins are strong antitumor compounds in murine leukemia animal models. The alkylated version luzopeptin A is three times more active than the semialkylated version luzopeptin B (luzopeptin C is inactive as antitumor in this *in vivo* model). Luzopeptin B has the same potency than echinomycin in this cancer model. Luzopeptin A is very active (100–300 times more than mitomycin) against *in vitro* leukemia, lung carcinoma, melanoma and sarcoma cell lines [17,116,117]. As antibacterial agent (*S. aureus*, *Streptococcus pyogenes*), luzopeptin A is weaker than echinomycins. However, luzopeptin A is very effective against *P. falciparum*, a parasite with an AT-rich genome (LD_50_ 9 × 10^−11^ M) [118]. Luzopeptin C and quinoxapeptin C are strong HIV-1 reverse transcriptase inhibitors, although these non-alkylated versions are weaker antitumor agents and weaker DNA bisintercalators [22,119].

## 5. Unnatural Derivatives

Diverse strategies for bisintercalators chemical synthesis have been described, as generation of triostin (and its *N*-demethylated derivative), luzopeptins, quinoxapeptins, sandramycin, quinaldopeptin, BE-22179, TANDEM, thiocoraline and echinomycin derivatives [33,89,120,121,122,123,124,125]. In many cases, these synthetic analogues have shed light into elucidation of the correct structure of the natural compounds.

Several echinomycin-like compounds have been synthesized by using glycophane scaffolds which have attached two QXCA chromophores. The goal here was to study their DNA-binding capacities [126]. Also, echinomycin analogues 1QN and 2QN have been semi-biosynthetically prepared, by feeding quinoline-2-carboxylic acid to *S. echinatus* cultures. One or two quinoline moieties are present in these two new compounds, in place of the natural quinoxaline chromophores. These changes affect the DNA sequence binding preferences, as compound 1QN still binds to 5′-CG rich DNA (as echinomycin does), whereas compound 2QN shows a strong preference for 5′-AT rich one. This demonstrates that chromophores also influence the sequence selectivity initially determined by the peptide backbone [127]. These experiments show the importance of chromophore moieties for DNA binding selectivity. Similar feeding experiments with other chromophores generated bis-6-chloro-, bis-6-bromo-, bis-7-chloro- and bis-3-amino-triostin. The bis-6-chloro and bis-6-bromo derivatives showed similar DNA-binding properties, whereas the bis-7-derivative showed a new marked preference for 5′-AT rich DNA [128]. In the case of TANDEM, several derivatives of this synthetic compound also exist, as for example one harboring l-Lys instead of the canonical l-Val at amino acid at positions E. [Lys^4^,Lys^8^]-TANDEM still shows a marked preference for 5′-AT rich DNA, but with higher affinity [129].

An array of sandramycin analogues with differences in the chromophore moieties has been created, showing that loss of the hydroxyl group at 3HQA enhances the antitumor activity by a factor of 10,000 [91]. 

Triostin and thiocoraline derivatives have been synthetized by using solid phase, in the latter case with amide macrolactone closure instead of thioester [130,131]. Azathiocoraline, for example, contains two amide linkages instead of the canonical thioester ones. This compound shows lower bioactivity because the presence of these two extra amide hydrogens changes the overall hydrogen bonding map of the molecule, avoiding DNA binding at good rates [132]. These authors generated also a further synthetic derivative, where these two amide linkages were methylated, giving rise to *N*Me-azathiocoraline. Bioactivity of *N*Me-azathiocoraline against several cancer cell lines (breast, lung, colon) was similar to thiocoraline levels, and with same sequence specificity. This demonstrates that it is possible to design and generate new derivatives with higher stability, still maintaining excellent DNA-binding capabilities and the original sequence specificity [133]. Also, *N*Me-azathiocoraline showed higher stability in human serum studies, with 23.1 h half-life, in contrast to 14.4 h half-life for thiocoraline. This is explained as those thioester linkages in thiocoraline molecule are a major cause of serum instability and enzymatic degradation [132]. FAJANU combinations of triostin, thiocoraline and luzopeptin have been created, in which the presence of heteroaromatic bicycles has been proved to be mandatory in order to retain bioactivity. Amide macrolactonization creates also more powerful compounds than their thioester versions [134,135]. Solid phase synthesis has been used for generation of oxathiocoraline, where the thioester linkage has been replaced by an ester one. Azathiocoraline also shows lower bioactivity than the parental compound, due to instability at these new ester linkages [136]. 

A three-plasmid system has been developed in order to generate diverse triostin/echinomycin derivatives in *E. coli* [27,137]. This system has been used to mutate the *N*-methyltransferase echinomycin NRPS domain responsible for *N*-methyl-l-Cys formation, and for omission of Ecm18 (responsible for thioacetal formation), generating TANDEM (*N*-desmethylated version of triostin), that was also synthetically generated before. TANDEM lacks antimicrobial bioactivity, therefore showing the importance of *N*-methyl moieties for this and probably also for its stability, as modified amino acids show higher resistance to proteases [123,138,139]. QXCA feeding to these *E. coli* strains greatly enhanced the final production levels [140]. 

In a nice combinatorial biosynthesis experiment, the hybrid compound ecolimycin was generated in *E. coli* by introducing diverse recombinant plasmids with genes coding for Ecm1-FabC initiation enzymes (which activate and bind QXCA), Ecm6 enzyme (which contains the first two NRPS modules (d-Ser, l-Ala)) and Swb17 enzyme (which contains last two NRPS modules from SW-163 (*N*-methyl-l-Cys, *N*-methyl-norcoronamic acid)). Generation of ecolimycin needed the feeding to *E. coli* with the rare precursor norcoronamic acid, as its biosynthesis is not present in this enterobacterium [27]. Ecolimycin was active against *Bacillus subtilis* at a similar level to SW-163C. This nice experiment proves the modularity of bisintercalator pathways and the promiscuity of NRPS modules in this family of compounds, opening the door to the rational generation of new unnatural derivatives. 

## 6. Conclusions

The current structural diversity of bisintercalator natural antitumor compounds, together with the proved promiscuity showed by some of their NRPS modules, as well as by tailoring enzymes, allows nowadays the rational design of novel combinatorial biosynthesis derivatives with potential enhanced bioactivities as antitumor, antiviral or antibacterial agents. This is specially favored by recent heterologous biosynthesis of some of these molecules in *E. coli*. Generation of new compounds is also feasible, as in recent years, huge efforts have allowed the design of new and interesting chemical approaches that are able to expand the still limited natural diversity of these bioactives. 
